# Using Quality Improvement to Improve Identification and Documentation of Malnutrition in Hospitalized Pediatric Patients

**DOI:** 10.1097/pq9.0000000000000504

**Published:** 2022-03-30

**Authors:** Shea T. Osburn, Mary Towne-Merritt, Roberta Baranda, Rhonda M. Keosheyan

**Affiliations:** From the Valley Children’s Hospital, Madera, Calif.

## Abstract

Recent publications have highlighted the importance and impact of recognizing malnutrition in hospitalized children. After noting that patients with malnutrition frequently went unrecognized in our facility, we implemented an interprofessional intervention comprising hospital medicine physicians, dietitians, and the clinical documentation improvement team to improve recognition and documentation of malnutrition in these patients, thereby facilitating earlier intervention.

**Methods:**

We implemented three separate plan-do-study-act cycles to improve the identification and documentation of malnutrition among patients hospitalized at our facility. The cycles consisted of identifying malnutrition using z-scores, educating providers, and implementing smart text within the medical record to help with consistent documentation. In addition, real-time communication between the disciplines (nutrition services, clinical documentation improvement providers, and hospitalists) was also employed to improve documentation quality. After completing the plan-do-study-act cycles, charts were reviewed to evaluate the nutritional interventions received.

**Results:**

Baseline data revealed that only 13% of patients with z-scores indicative of malnutrition were identified as such in attending physicians’ documentation. Upon implementation of our plan-do-study-act cycles, documentation of these patients increased to greater than 64%. Patients with documented malnutrition received nutritional interventions at least 81% of the time, increasing from 35% at baseline.

**Conclusion:**

Our findings demonstrate that an interprofessional approach can dramatically enhance the identification and documentation of malnutrition in hospitalized children, leading to earlier intervention.

## INTRODUCTION

Pediatric malnutrition is a frequently encountered problem in hospitalized pediatric patients. We aimed to improve awareness and intervention through a Quality Improvement project. We utilized a prospective approach to improving documentation and intervention for malnutrition using an inter-professional team and standard qualitative metrics.

While malnutrition is typically addressed in the outpatient setting in developed countries, recent data have shown that poor nutrition can cause significant morbidity among hospitalized children.^1^ Malnutrition has been associated with increased duration of mechanical ventilation among children with bronchiolitis in an intensive care unit,^2^ and increased rates of infection after surgery.^3^ Early and consistent recognition of malnutrition in pediatric patients allows for earlier intervention, decreased length of stay, and prevention of iatrogenic malnutrition during the hospital stay.^4^

Malnutrition can refer to undernutrition or over-nutrition and is a state in which a deficiency or excess of energy, protein, and other nutrition causes measurable adverse effects on a body and growth.^5^ For our study, malnutrition refers to undernutrition only. In the pediatric hospital setting, we typically define malnutrition severity using z-scores. Calculated z-scores are a statistical measurement using weight-for-height, height-for-age, and weight-for-age values compared with the mean values for that age group. Based on this calculation, the severity can be mild, moderate, or severe if malnutrition is present.^1,6,7^

Children are particularly vulnerable to malnutrition because they are dependent on adults for their care and wellbeing. As many as 24% of hospitalized children in developed countries suffer from malnutrition.^1^ Despite this knowledge, the under-recognition of this comorbid condition continues to be prominent in these patients. At our institution, our baseline data revealed that only 13% of patients discharged from the hospital medicine service with z-scores less than −1 had malnutrition documented in their medical records. Additionally, only 35% of these patients received nutritional interventions at discharge.

Prior studies have suggested that screening for malnutrition at the beginning of an illness allows assessment of current nutritional status and facilitates early detection of subsequent nutritional deterioration related to illness.^8^ To intervene on all identifiable patients at risk for worsening of their nutritional status, we included patients with mild to severe malnutrition in our study. However, even the mild population is at risk for worsening of their malnutrition with acute illness. Therefore, with the plan to encompass as many at-risk patients as possible, we designed an interprofessional approach to enhance the consistency of malnutrition recognition and promote documentation of this condition in the assessment and plan section of the patient’s progress note. Via this intervention, our specific aim was to increase documentation of malnutrition for patients with z-scores less than −1 on the hospital medicine service from 13% to 64%. Our clinical documentation improvement team recommended this increase in fifty percentage points.

## METHODS

### Study Setting

Our hospital is a free-standing children’s hospital with 196 acute care beds. The hospital covers 1.6 million pediatric lives in the central valley of California. It provides primary and specialized pediatric services and is a safety-net hospital that combines teaching with a mission to serve many low-income patients in a rural setting. The pediatric hospital medicine division cares for approximately 35%–50% of the acute admissions throughout the year. The patient population encompasses general pediatrics, technology-dependent children, and surgical and subspecialty co-management. Our hospital medicine service documents clinical care entirely in an electronic format. Our electronic health record allows for phrases, paragraphs, plans, and other frequently used elements of physician documentation to be saved and easily accessed by different users by utilizing a series of specific keystrokes that implement the entire text to capture more comprehensive and standardized documentation for our patients.

### Study Population

We included all acute care patients over 2 weeks of age admitted to the hospital medicine service over 4 months (July 1–October 31) with an identified z-score less than −1 for weight/height during defined PDSA cycles in this project based on data extracted from our electronic medical record. We excluded patients with no documented z-scores and those less than 2 weeks of age.

Our in-house dietitians screen and stratify patients at admission using Peditools.org.^9^ They also calculate z-scores as part of their patient assessment. All patients identified as high-risk undergo a complete dietary assessment, including a z-score, within 24 hours.

### Interventions

**Fig. 1.**
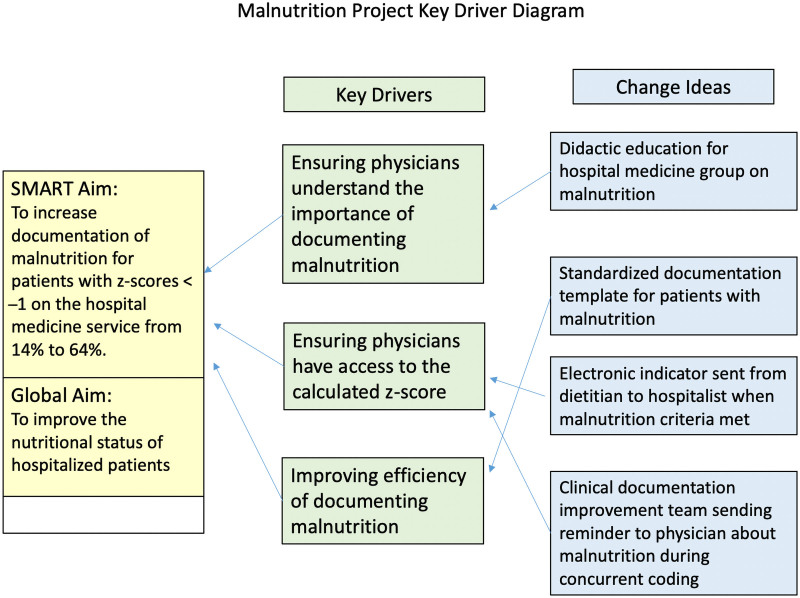
Key driver diagram. A visual display of the key drivers impacting change for this project.

The project team consisted of an administrative sponsor, hospital medicine leadership, nutritional services, data experts, an information technologist, a case management leader, and a quality and safety champion. We determined the key drivers for this improvement project: physician understanding of the importance of documenting malnutrition, reliable access to the calculated z-score, and facilitating the physician documentation (See key driver diagram, Fig. 1.)

**Fig. 2.**
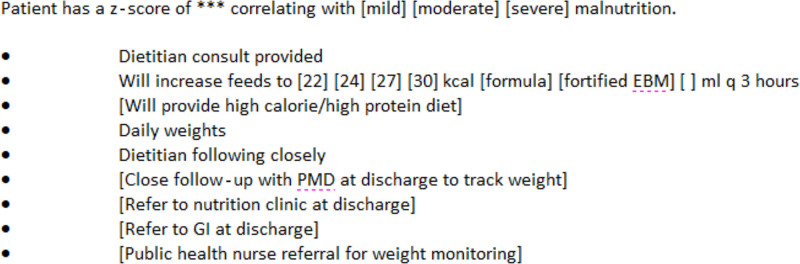
Example of the standardized documentation template implemented in this study. The template designed and utilized at our facility to pull z-scores for each patient and provide options for interventions. Physicians were allowed to choose interventions at their discretion.

Before our interventions, we collected 6 months of baseline data. These data revealed an overall 13% compliance with the documentation of malnutrition. After a period of planning and organizational approval, we completed three plan-do-study-act (PDSA) cycles.^10^ We designed short, 2-week cycles to make rapid changes in practice. These cycles were not done at a regular interval due to resource limitations and organizational restraints. Because of the varied stakeholders, studying the cycle and planning the subsequent intervention required additional planning between the PDSA cycles. The first cycle comprised education of the entire hospital medicine group and implementing a standardized template for documenting malnutrition in the assessment and plan section of the medical record. Education included a 1-hour live presentation emphasizing the importance of recognizing and documenting malnutrition and instructions on accessing and utilizing the new document template. Additionally, we sent follow-up email reminders to the group every 1–2 weeks during the entire project. Finally, we collaborated with our Informatics team and designed a standardized documentation template to automatically pull in z-scores and provide interventions (Fig. 2). The physician could then utilize all, some, or none of the smart text options, as desired.

The second PDSA cycle consisted of an electronic indicator sent by nutritional services that a hospital medicine patient had a z-score less than −1. The dietitians sent a message directly to the attending physician upon completing the patient’s full nutritional assessment.

The final PDSA cycle involved a second reminder sent by our clinical documentation improvement team while reviewing concurrent coding records. Specifically, if a patient was noted to have malnutrition by z-score but lacked the documentation, an electronic notification was sent to the attending physician for that patient as a reminder to include malnutrition in the assessment and plan sections of the progress note.

### Measures

We developed this study based on the Model for Improvement^11^approach utilizing PDSA cycles, and it was a prospective quality improvement effort. We evaluated the project using process, outcome, and balancing measures while monitoring data over time. The primary measure was the percentage of hospitalist service patients with a z-score less than −1 who had malnutrition documented. The numerator consisted of the number of patients with appropriate documentation, and the denominator represented all patients on the hospitalist service with a z-score less than −1. Balancing measures included ensuring that lengths of stay were not increased and that the dietitians’ and clinical documentation specialists’ workloads were not significantly impacted. Their direct supervisors reconciled this measure by ensuring that each team could support the same number of patients each day and did not require an increase in overtime.

The primary process measure consisted of reviewing the percentage of patients with a low z-score documented in the patient notes’ assessment and plan section. In addition, we monitored documentation weekly during the PDSA cycles, and updates were provided to the hospital medicine team at monthly meetings and more frequently via e-mail to motivate continued participation.

Finally, as an outcome measure, we reviewed all charts with malnutrition documented during the study period to determine if a nutritional intervention occurred during the hospitalization. Nutritional interventions included increased caloric density of feedings, feeding supplementation, increased feeding volumes, nasogastric or gastrostomy tube placement, and/or specialist involvement in the patient’s care. We included only patients with these interventions continued on discharge from the hospital, as short-term interventions during the hospital stay may have been related to an acute clinical issue, such as NG tube feedings while they recover from encephalitis or botulism. If the patient had malnutrition that the clinician believed was relevant long-term, those nutritional interventions would have been continued at discharge.

### Analysis

**Fig. 3.**
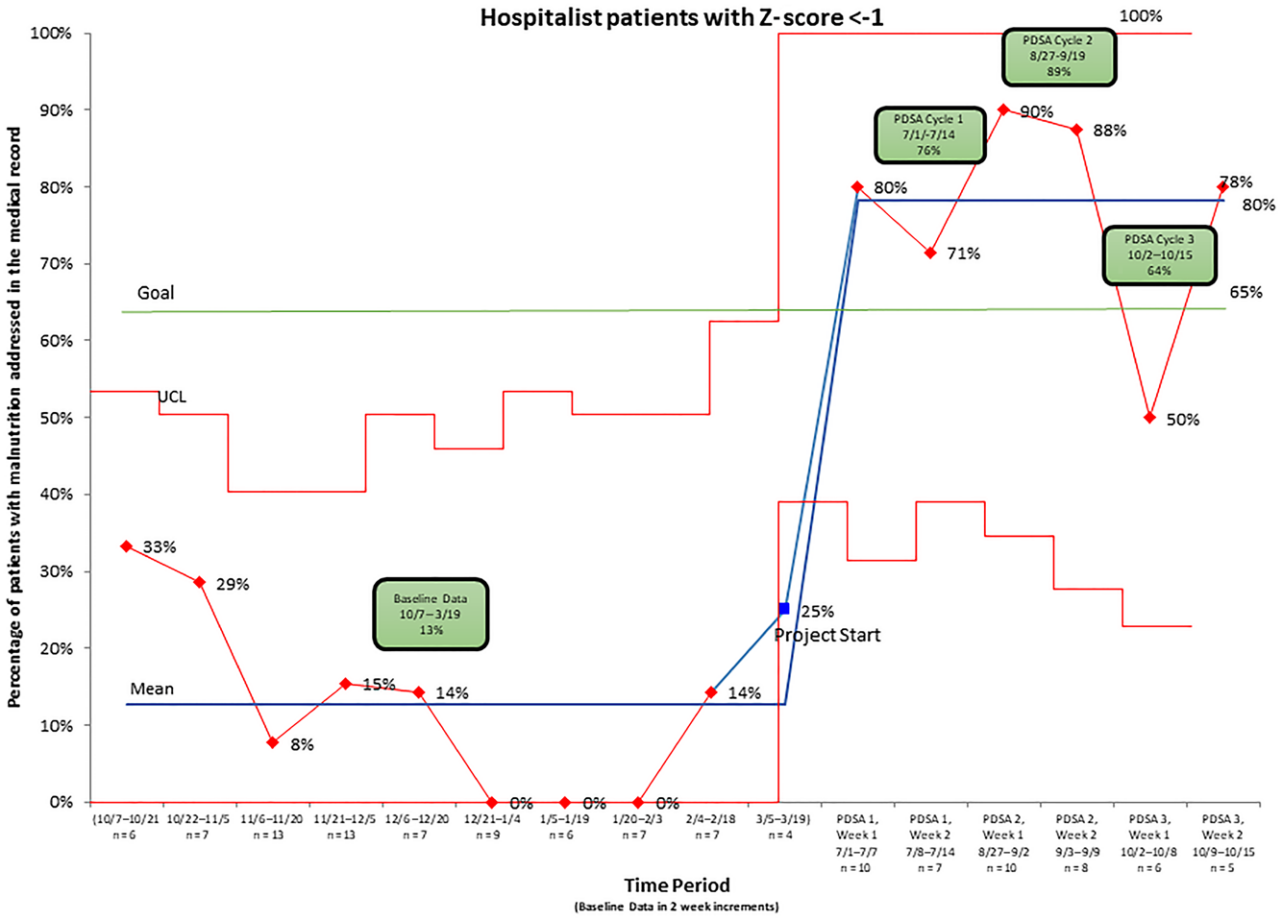
Control chart depicting the percentage of charts with malnutrition documented during baseline data collection and the intervention period. The diagram represents the percentage of charts with documented malnutrition during the baseline data collection period and the three PDSA cycles.

Before starting this project, we obtained baseline data by reviewing discharged patient records from the hospital medicine service over 6 months. The percentage of patients who had malnutrition appropriately documented in the medical record by the physician was compared to all hospital medicine patients with a z-score less than −1, as determined by nutritional services. Following each 2-week PDSA cycle, we repeated data analyses and compared results with the goal. We then plotted process measures on a statistical process control chart (Fig. 3) to understand if our changes resulted in improvement. Straight percentages were determined to supply data for the chart. After completing the PDSA cycles, we reviewed the patients’ charts with documented malnutrition to determine if our team provided nutritional interventions for the patient and created a table to display the outcome measure (Table 1). As evidenced by our statistical process control, there was a significant increase in the documentation of malnutrition during the PDSA cycles resulting in a centerline shift, verifying that our interventions resulted in improvement.^12^

**Table 1.** Table Representing Patients with Malnutrition Documented during the PDSA Cycles and the Percentage with Nutritional Interventions

	PDSA 1	PDSA 2	PDSA 3
Age (y)			
Mean	4.5 y	5 y	8 r
Median	2 y	3 y	2 y
Z-Score			
Mean	−2.66	−2.56	−4.625
Median	−2.24	−2.25	−3.9
No. patients in cycle with documented malnutrition	13	16	7
Percentage of patients with nutritional intervention when malnutrition documented	92%	81%	100%

### Ethical Considerations

The Performance Improvement Committee approved this project at our institution per our Institutional Review Board policy. The project was determined to be “Not Human Subjects Research,” as it does not meet the federal definition of research under 45 CFR 46.102(d). It was determined to be a Quality Improvement Project and deemed not subject to further Institutional Review Board review.

## RESULTS

Figure 3 outlines the percentage of patients with malnutrition in the medical record throughout the study period. Our baseline data were plotted in 2-week increments to be consistent with our PDSA cycles. During 2/19–3/4, only 1 patient was found to have malnutrition, and it was documented. Because there was only one patient identified during that entire 2-week period, it was determined to be a special cause event and removed from the calculation of the baseline dataset. Notably, after the first PDSA cycle, the percentage of patients with a z-score less than −1 with documented malnutrition rose from the baseline of 13%–76%. Following the second cycle, the percentage further improved to 89%. However, there was a drop in documentation in the third PDSA cycle to 64% due to low malnutrition documentation in the first week of that PDSA cycle. We cannot explain this drop; however, as one data point should not determine success in Quality Improvement projects^13^ and the percentage was still much higher than during the baseline period and rebounded the next week, we do not believe it represents an inherent problem with the project. In the first PDSA cycle, 92% of patients received a nutritional intervention. In the subsequent PDSA cycles, it was 81% and 100%, respectively. This result was a substantial improvement over the 35% of patients who received interventions during the baseline period.

Concerning balancing measures, the leadership of the clinical documentation improvement team and our nutritional services team reported no impact on the number of patients serviced by their teams. Neither team required additional overtime during the study period. There was no overall increased length of stay of acute care patients at our hospital during the study period.

## DISCUSSION

Recent publications highlight the under-recognition of malnutrition in hospitalized children.^1,4,6,14^ It is a common co-morbid condition that impacts care delivery, length of stay, and long-term outcomes.^8,15^ Current data suggest that although this condition is traditionally addressed in the outpatient setting, acute care hospitalization is an appropriate time to initiate malnutrition interventions.^16^ Our ultimate goal is to impact this patient population by early identification to initiate appropriate intervention. Although the number of patients in each cycle was relatively small, longer cycles would have captured more patients but potentially prevented rapid improvement.

Using the Process Improvement Model for Healthcare Improvement,^11^ we developed and implemented PDSA interventions sequentially, allowing for rapid improvement over a short period. In addition, this project was an interprofessional intervention, bringing key stakeholders together to impact change. By each discipline making small changes in their daily practice, we dramatically improved the number of patients identified with malnutrition without significantly impacting workflow or time management.

As a novel approach to obtain physician buy-in and active participation, we embarked on pairing this project with maintenance of certification. As a result, we experienced sustained engagement and even enthusiasm from the physicians ultimately responsible for malnutrition documentation by incorporating needed maintenance of certification credit. Also, we required completion of learning modules on basic improvement science to obtain maintenance of certification credit, generating a general interest in performance improvement within our group. We believe this was instrumental in achieving the rapid improvement we achieved with this project.

In addition to interprofessional engagement, sustainability is vital for long-term success in identifying patients with malnutrition. We attained buy-in from the end-users by creating a smart text script that was easy to use and automatically pulled in z-scores and possible interventions. The changes to the workflow implemented during this project have become ingrained as new physicians, dietitians, and clinical documentation improvement specialists join the staff. Despite a drop in documentation compliance in the third PDSA cycle, the change from baseline was substantial. By implementing a standardized, easy-to-use template, we demonstrated that most patients received interventions to address their malnutrition upon discharge.

There were several limitations to this project. First, patients may not have received a formal nutritional assessment and z-score generated before discharge. However, our dietitians screen everyone within 24 hours. Therefore, only patients with short lengths of stay would not have z-scores. Also, incorrectly measured height could result in an inaccurate z-score. Moreover, z-scores could potentially be influenced by transcription errors while entering the patient’s height and weight into the medical record or the dietician’s screening tool. Lastly, we included only patients on the hospital medicine service in this study.

The next steps should include education and engagement of all admitting physicians at our institution to identify all patients with malnutrition. Malnutrition is known to be associated with poverty. Our hospital serves an impoverished population in the central valley of California, making early identification key to impacting the overall health of these patients.^17^ Additional next steps should include a review of z-scores after discharge in partnership with the patient’s primary care provider to see if we can sustain a long-term impact on the nutritional status of patients.

We conducted this project within a single free-standing children’s hospital. The resources and collaboration present at other institutions, including community-based smaller pediatric units, may make implementing a project like this more difficult. However, an ever-increasing population of complex pediatric patients with many comorbidities, including malnutrition, and early identification of problems that impact growth can have enormous health benefits.^5,14,18^ It would therefore benefit all providers of pediatric health care to have processes in place that identify these patients as early as possible, to then allow for intervention.^8,19–24^

## SUMMARY

A quality improvement project on recognizing malnutrition in pediatric patients in the inpatient setting can lead to real-time interventions to address their malnutrition.

## DISCLOSURE

The authors have no financial interest to declare in relation to the content of this article.

## ACKNOWLEDGMENTS

The authors would like the thank Christine White, MD, MAT, for her aid in manuscript development, and Raed Khoury, MA, MPH, Jasmine Fernandez, BS, and Erica Lane, MHA, for help with data analysis and chart development.
